# Establishment of *lal-/-* Myeloid Lineage Cell Line That Resembles Myeloid-Derived Suppressive Cells

**DOI:** 10.1371/journal.pone.0121001

**Published:** 2015-03-25

**Authors:** Xinchun Ding, Lingyan Wu, Cong Yan, Hong Du

**Affiliations:** 1 Department of Pathology and Laboratory Medicine, Indiana University School of Medicine, Indianapolis, IN, United States of America; 2 IU Simon Cancer Center, Indiana University School of Medicine, Indianapolis, IN, United States of America; 3 The Center for Immunobiology, Indiana University School of Medicine, Indianapolis, IN, United States of America

## Abstract

Myeloid-derived suppressor cells (MDSCs) in mouse are inflammatory cells that play critical roles in promoting cancer growth and metastasis by directly stimulating cancer cell proliferation and suppressing immune surveillance. In order to facilitate characterization of biochemical and cellular mechanisms of MDSCs, it is urgent to establish an “MDSC-like” cell line. By cross breeding of immortomouse (simian virus 40 large T antigen transgenic mice) with wild type and lysosomal acid lipase (LAL) knock-out (*lal-/-*) mice, we have established a wild type (HD1A) and a *lal-/-* (HD1B) myeloid cell lines. Compared with HD1A cells, HD1B cells demonstrated many characteristics similar to *lal-/-* MDSCs. HD1B cells exhibited increased lysosomes around perinuclear areas, dysfunction of mitochondria skewing toward fission structure, damaged membrane potential, and increased ROS production. HD1B cells showed increased glycolytic metabolism during blockage of fatty acid metabolism to fuel the energy need. Similar to *lal-/-* MDSCs, the mTOR signal pathway in HD1B cells is overly activated. Rapamycin treatment of HD1B cells reduced ROS production and restored the mitochondrial membrane potential. HD1B cells showed much stronger immunosuppression on CD4^+^ T cell proliferation and function *in vitro*, and enhanced cancer cells proliferation. Knockdown of mTOR with siRNA reduced the HD1B cell ability to immunosuppress T cells and stimulate cancer cell proliferation. Therefore, the HD1B myeloid cell line is an “MDSC-like” cell line that can be used as an alternative *in vitro* system to study how LAL controls various myeloid cell functions.

## Introduction

Myeloid-derived suppressor cells (MDSCs) are myeloid progenitors that are blocked to further differentiate into granulocytes, macrophages, and dendritic cells at various pathogenic conditions [1,2]. In mice, MDSCs are broadly defined as CD11b^+^Gr-1^+^ cells. MDSCs in the tumor microenvironment have been suggested to have a causative role in directly stimulating cancer cell proliferation and promoting tumor-associated immune suppression. Since MDSCs may serve as a target for preventing tumor growth and metastasis, there is a need to establish *in vitro* “MDSCs-like” cell lines to facilitate MDSCs studies at the cellular and molecular levels.

Fatty acid metabolism supports both the biosynthetic and bioenergetic requirements of cell proliferation and survival. Lipids are essential components of plasma and organelle membranes, and can function as secondary messengers for signal pathways. In addition to glycolytic metabolic pathway, free fatty acids oxidation (FAO) also serves as an important metabolic fuel for energy production (e.g., ATP) on the mitochondrial electron transportation chain. Lysosomal acid lipase (LAL) is an essential enzyme that hydrolyzes cholesteryl esters (CE) and triglycerides (TG) to generate free fatty acid (FA) and cholesterol in lysosomes. Lack of LAL in humans leads to two human lipid storage diseases, Wolman disease (WD) and CE storage disease (CESD). Increased CD14^+^CD16^+^ and CD14^+^CD33^+^ cells have been linked to heterozygote carriers of LAL mutations in humans [3]. CD14^+^ CD16^+^ and CD33^+^ are the markers used for human subset of MDSCs identification [4]. In mice, lack of LAL in genetically ablated knockout mice (*lal*-/-) shows systemic expansion of MDSCs, which influences the tissue microenvironment and contributes to local pathogenesis [5–7] [8,9] [10]. *Lal-/-* MDSCs directly stimulate cancer cell proliferation [11], and suppress T cell proliferation and impair T cell function [12]. Myeloid-specific expression of human LAL in *lal-/-* mice reverses tissue inflammation, MDSCs infiltration, and corrects malformation and dysfunction of MDSCs [13,14].

In order to fully understand the functional role of LAL in MDSCs development, the Affymetrix Genechip microarray assay was performed. The gene profile showed upregulation of metabolic enzyme genes in glycolysis and citric acid cycle in association with over-activation of the mTOR signaling pathway in *lal-/-* MDSCs in which their fatty acid generation is blocked [15]. The mTOR signaling regulates nutrient energy and metabolism, controls cell growth and division [16]. The mTOR signaling pathway plays a critical role in modulating immune functions [17]. Inhibition of mTOR pharmacologically or by siRNA knockdown reduces *lal-/-* MDSCs abilities to stimulate cancer cell proliferation and to suppresses T cell proliferation and function [11,18].

Mitochondria fission (fragment or dot shape) and fusion (filamentous) play critical roles in maintaining functional mitochondria when cells are under metabolic or environmental stress [19]. Studies have reported that mitochondria fission and fusion respond to cellular triglyceride accumulation [20]. Since the mTOR pathway is highly activated, mitochondria membrane potential is damaged, and the ROS level is elevated in *lal-/-* MDSCs [18], it is essential to examine the mitochondria fission and fusion in these MDSCs like cells.

In this report, immortalized wild type *lal*+/+ HD1A and *lal*-/- HD1B myeloid lineage cell lines were established from wild type and *lal-/-* mice that were crossbred with Immortomouse expressing a temperature-sensitive version of simian virus 40 large T antigen. The key characters of MDSCs were analyzed in HD1A and HD1B cell lines. HD1B cells showed higher proliferation than that of HD1A cells. This is accomplished by high consumption of glucose oxidation in the mitochondria to compensate the deficiency of FAO. Similar to its primary precursor *lal*-/- MDSCs, *lal*-/- HD1B myeloid cells *in vitro* showed stronger immunosuppression on T cells, and stronger stimulation on cancer cell proliferation compared with its wild type counterpart HD1A cells. At the cellular level, HD1B cells showed characteristics of *lal-/-* MDSCs, including over-activation of the mTOR signaling pathway, increased production of reactive oxygen species (ROS), arginase activity, and damaged membrane potential. At the subcellular level, the mitochondrial organization of HD1B cells morphologically showed more fission structure in association with down-regulation of pro-fusion protein Opa1 and phosphorylated activation of pro-fission protein Drp1, while the mitochondrial organization of wild type HD1A cells showed more fusion structure. Establishment of these cell lines will not only facilitate elucidation of cellular and molecular mechanisms that are involved in MDSCs malformation and malfunction, but also provide an *in vitro* system to screen drugs for pharmacological and immune therapy purposes.

## Materials and Methods

### Ethics Statement

All scientific protocols involving the use of animals have been approved by the Institutional Animal Care and Use Committee of Indiana University School of Medicine and followed guidelines established by the Panel on Euthanasia of the American Veterinary Medical Association. Animals were housed under Institutional Animal Care and Use Committee-approved conditions in a secured animal facility at Indiana University School of Medicine.

### HD1A and HD1B myeloid cell line establishment from isolation of immortalized mouse myeloid lineage cells

Peritoneal macrophages were collected from wild type and *lal-/-* male mice that had been crossbred with Immortomouse (Charles River Laboratories) expressing a temperature-sensitive version of simian virus 40 large T antigen from an IFN-γ inducible promoter [21]. Cell suspensions were obtained by peritoneal lavage with 8 ml of PBS, washed, and cells were cultured at 33°C in RPMI medium 1640 supplemented with 10% FBS, antibiotics, and 5 units/ml IFN-γ. After 10 passages, IFN-γ was omitted from the medium.

### Living cell lysosome staining

HD1A and HD1B cells were grown in 24-well plates to the desired confluence. The medium were replaced with pre-warmed (37°C) LysoTracker Red DND-99 probe (50nM, Molecular Probes)-containing medium for 1 hour. Cells were replaced with fresh medium, and fluorescent signals were examined under the Nikon ECLIPSE Ti inverted fluorescence microscope.

### Glucose and pyruvate measurement

The concentration of glucose and pyruvate was measured by the glucose assay kit and pyruvate assay kit (Sigma) respectively according the manufacturer’s instruction. Briefly, HD1A or HD1B cells were washed with PBS before being harvested. The cell pellets were added with pre-warmed water and headed in the 70°C water bath for 10 minutes. After spinning down the cell lysates, for glucose measurement the supernatants were incubated with glucose assay reagent for 15 minutes at room temperature and measured absorbance at the 340 nm. For pyruvate measurement, the supernatants were incubated with pyruvate assay buffer, pyruvate probe solution and pyruvate enzyme mixture for 30 minutes at room temperature and measured absorbance at the 570 nm.

### Aconitase activity assay

The aconitase activity was measured by the aconitase activity assay kit (Sigma) according the manufacturer’s instruction. Briefly, HD1A or HD1B cells were washed with PBS before being harvested. Cells were lysed in the ice-cold assay buffer. After centrifugation, the aconitase activation buffer was added into supernatant and incubated on ice for 1 hour, followed by adding Enzyme Mix, assay buffer and the substrate at 25°C for 30 minute. After addition of developer to the incubation mixture at 25°C for 10 minute, the reactions were measured at the 450 nm absorbance. One unit of the aconitase activity is the amount of enzyme that isomerize 1.0 μmole of citrate to isocitrate per minute at pH 7.4 at 25°C.

### Living cell mitochondrial staining

HD1A and HD1B cells were grown in 24-well plates to the desired confluence. The medium were replaced with pre-warmed (37°C) MitoTracker Green FM (100 nM, Molecular Probes)-containing medium for 1 hour. Cells were replaced with fresh medium, and fluorescent signals were examined under the Nikon ECLIPSE Ti inverted fluorescence microscope.

### Immunofluorescence

HD1A and HD1B cells were fixed for 15 min in 4% paraformaldehyde, and permeabilized for 10 min in 0.02% Triton X 100. After washing, cells were blocked with 5% normal goat serum in 1 x PBS for 1 hour followed by incubation of primary goat anti-LAMP1 antibody (1:200, Santa Cruz) overnight. Cells were then incubated with secondary donkey anti-goat antibody conjugated with Cy3 (1:1000, Jackson ImmunoResearch) for 1 hour, and co-stained with DAPI. Fluorescent signals were examined under the Nikon fluorescence microscope.

### Western blot

HD1A and HD1B cells were lysed in the Cell Lytic M mammalian cell lysis/extraction buffer (Sigma-Aldrich) according to the manufacturer’s instruction. Protein samples were fractionated on a Novex 4–20% Tris-Glycine Mini Gel (Invitrogen). After transferring to the polyvinylidene difluoride membrane (Bio-Rad), the membrane was blotted with 5% nonfat dry milk in 1x PBS, and incubated with rabbit anti-p-S6 (Ser235/236), anti-S6, anti-p-Drp1 (Ser616), anti-Drp1, and anti-actin primary antibodies (from Cell Signaling), or rat anti-LAMP1, rabbit anti-Opa1 antibodies (from Santa Cruz). Following incubation with the secondary antibody that conjugated with horse radish peroxidase, proteins were visualized with chemiluminescent substrate (Thermo Scientific) under ChemiDox MP Image System.

### ROS measurement

HD1A and HD1B cells were treated with solvent, rapamycin (40 nM), or PP242 (40 nM) for 60 minutes. Cells were washed and stained with 2 μM of 2',7'-dichlorodihydrofluorescein diacetate stained (DCFDA, Invitrogen) which is nonfluorescent until the acetate group is removed by intracellular esterases and oxidation occurs within the cell (Invitrogen). The incubation was at 37°C for 20 minutes, followed by washing with cold PBS. The ROS level was measured by flow cytometry.

### Arginase activity measurement

HD 1A or HD1B cells were lysed for 30 min at room temperature with 50 μl 0.1% Triton X-100 PBS containing 5 μg pepstatin, 5 μg aprotinin, and 5 μg antipain protease inhibitors per ml. Subsequently, 50 μl 10 mM MnCl_2_ and 50 μl 50 mM Tris-HCl (pH 7.5) were added, and the enzyme was activated by heating at 56°C for 10 min. Arginine hydrolysis was conducted by incubating the lysate (100 μl) with 100 μl 0.5 M L-arginine (pH 9.7) at 37°C for 60–120 min. The reaction was stopped with 400 μl H_2_SO_4_ (96%)/H_3_PO_4_ (85%)/H_2_O (1:3:7, v/v/v). The urea concentration was measured at 540 nm after addition of 25 ml 9% α-isonitrosopropiophenone (dissolved in 100% ethanol), followed by heating at 95°C for 45 min and 10 min at room temperature in the dark. One unit of enzyme activity is defined as the amount of enzyme that catalyzes the formation of 1 μmol urea per minute.

### T cell proliferation and lymphokine release assays *in vitro*

Freshly isolated wild type CD4^+^ T cells from the spleen were labeled with carboxyfluorescein diacetate succinimidyl diester (CFSE, Molecular Probes) (1 μM in PBS) at room temperature for 5 min, and resuspended in complete medium for 20 min. CD4^+^ T cells were spun down and cultured in 96-well flat-bottom plates coated with anti-CD3 mAb (2 μg/ml) and anti-CD28 mAb (5 μg/ml) for 4 days in the presence or absence of HD1A or HD1B cells at 37°C. The ratio between HD1A or HD1B cells and CD4^+^ T cells was 1:30. Cells were harvested and stained with APC-labeled anti-CD4 mAb (eBiosciences). Proliferation of CD4^+^ T cells was evaluated as CFSE dilution by FACS. To measure T cell secreting lymphokines, OptEIA ELISA kits for IL-2, IL-4 and IFNγ were used according to the manufacturer’s instruction (BD BioScience).

### HD1A and HD1B cell surface marker staining

HD1A and HD1B cells were harvested and stained with anti-CD11b-PEcy7 and anti-Ly6G-Apccy7 for flow cytometry analysis.

### mTOR knockdown by siRNAs

HD1A and HD1B cells were transfected with mTOR—specific or scrambled control siRNAs (final concentration 25 nM) according to the manufacturer’s protocol (Dharmacon, Lafayette, CO). After 48 hours incubation, cells were washed and co-cultured in wells with CFSE-labeled wild type CD4^+^ T cells (1:30) for T cell proliferation (96 hours) and lymphokine release study (48 hours), or lysed to test mTOR signaling pathway protein expression, or co-cultured with B16 or LLC cancer cells, or labeled for BrdU incorporation study.

### Mitochondrial membrane potential assay

HD1A and HD1B cells were grown in 24-well plates to the desired confluence. Cells were treated with solvent (DMSO, 0.1%), rapamycin (40 nM), or PP242 (40 nM) for 1 hour, or NAC (100 μM, Sigma-Aldrich), or Tempol (10 μM, Sigma-Aldrich) overnight. Treated cells were replaced with the pre-warmed (37°C) medium containing JC1 (5 μM, Molecular Probes) for 1 hour. Labeled cells were replaced with fresh medium and examined under the Nikon inverted fluorescence microscope.

### BrdU incorporation

For cell proliferation analysis, HD1A and HD1B cells were grown in 24-well plate to the desired confluence. BrdU (BD Biosciences) was added at a final concentration of 10 μM in cell culture medium for 1 hour. Cells were harvested and washed twice with PBS. Cells were fixed and permeabilized with BD Cytofix/Cytoperm buffer, then incubated with DNase I and washed again followed by staining with fluorescent anti-BrdU antibody before analysis by flow cytometry.

### Cancer cell proliferation *in vitro*

B16 melanoma or LLC cancer cells were harvested and labeled with CFSE (1 μM in PBS) at room temperature for 5 min. Labeled cancer cells were resuspended in complete medium for 20 min, spun down, and co-cultured with HD1A or HD1B cells (1:5, 3 x10^4^ cells per well of 24-well plate). After culture for 3 days, the cells were harvested and analyzed on the LSR II to determine cancer cell proliferation by gating CFSE labeled cells.

### Cancer cell growth in vivo

B16 melanoma cells (2 X 10^5^) were mixed with HD1A or HD1B cells (2 X 10^5^) and injected subcutaneously at left or right flank sites of C57BL/6 or FVB/N mice. The tumor sizes were measured 14 days post-injection with calipers. The tumor volumes were determined using the formula: (length X width^2^) /2 [11]. At the end of the experiment, the animals were euthanized.

### Real-Time PCR

Total RNAs from HD1A or HD1B cells were purified using the Qiagen total RNA purification kit (Qiagen). cDNAs were generated by SuperScript III (Invitrogen). Real-Time PCR for *CD36*, *CPT1a*, *CPT1b*, *CPT1c*, *Foxo3*, *Glut1-13*, *IDO1*, *IDO2*, *SIRT1* and the housekeeping gene β-*Actin* was performed on a StepOnePlusReal-Time PCR System (Applied Biosystems) using Power SYBR Green PCR Master Mix (Applied Biosystems) according to the manufacturer’s protocol. The (2^-˙ΔΔCt^) algorithm was used to determine the relative gene expression.

## Results

### Lysosome accumulation in *lal-/-* macrophage cell lines

**Fig 1:**
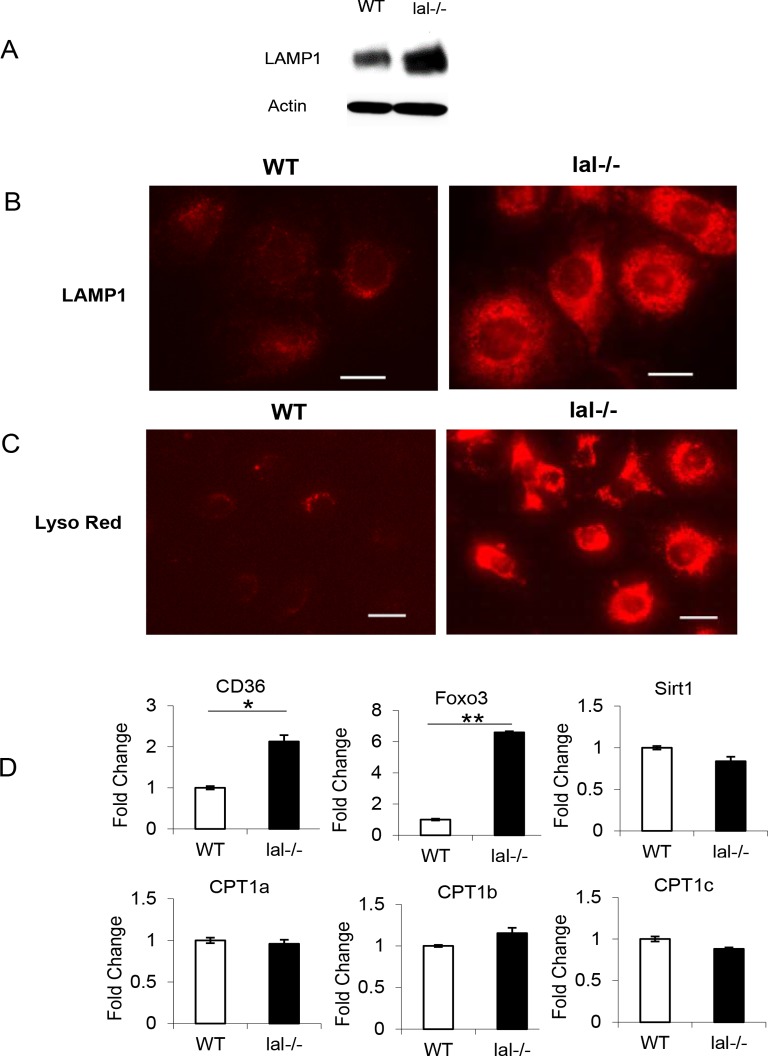
Increase of lysosome genesis and metabolic disorder in HD1B cells. (A) Western blot analysis of lysosome marker LAMP1 expression in wild type (HD1A) and *lal*-/- (HD1B) cells; (B) Immunofluorescence staining of LAMP1 in wild type (HD1A) and *lal*-/- (HD1B) cells. Bars = 20 μm; (C) LysoTracker Red DND-99 staining of live wild type (HD1A) and *lal*-/- (HD1B) cells. Bar = 25 μm. (**D**) Expression of CD36, Foxo3, Sirt1, CPT1, CPT2, CPT3 with the housekeeping gene β-Actin as internal control by Real-time PCR. The results are means ± SD from three independent experiments (n = 3), *, p< 0.05, **, p< 0.001.

To generate HD1A and HD1B cells, wild type and *lal*-/- mice were crossbred with Immortomouse (Charles River laboratory), which express a temperature-sensitive simian virus 40 T antigen under an INF-γ inducible promoter. HD1A and HD1B cell lines were established and passaged as described in Materials and Methods. As LAL is a lysosome localized enzyme, the lysosome numbers and localization were examined in HD1A and HD1B cells. Western blot analysis showed an increased LAMP1 (a marker for lysosome) expression level in HD1B cells compared with that of HD1A (Fig. 1A). Immunofluorescent staining of LAMP1 showed increased lysosomal numbers in HD1B cells around the perinuclear area (Fig. 1B). This was confirmed by another lysosome specific dye LysoTracker Red DND-99 staining (Fig. 1C). The malfunction of HD1B was assessed by several molecules that are involved in fatty acid uptake and function, including CD36, forkhead box O (FoxO3), and SIRT1. CD36 or FOXO3 expression was increased in HD1B cells compared with HD1A cells, while SIRT1 remained no change (Fig. 1D). We also investigated another important group of FA transporters, carnitine palmitoyl transferase (CPT1a, CPT1b and CPT1c) that transports long-chain FA into the mitochondria and are a rate limiting step of mitochondrial fatty-acid oxidation (FAO). None of these transporters showed expression changes in HD1B cells compared with those in HD1A cells (Fig. 1D), suggesting no increased activity of this pathway for FA transportation into the mitochondria during LAL dysfunction. Taken together, these results indicate that LAL deficiency increases lysosome genesis and the abnormal activities of fatty acid metabolism in HD1B myeloid cells.

### Glucose level, pyruvate level, aconitase activity, and GLUT expression in HD1A and HD1B cells

**Fig 2:**
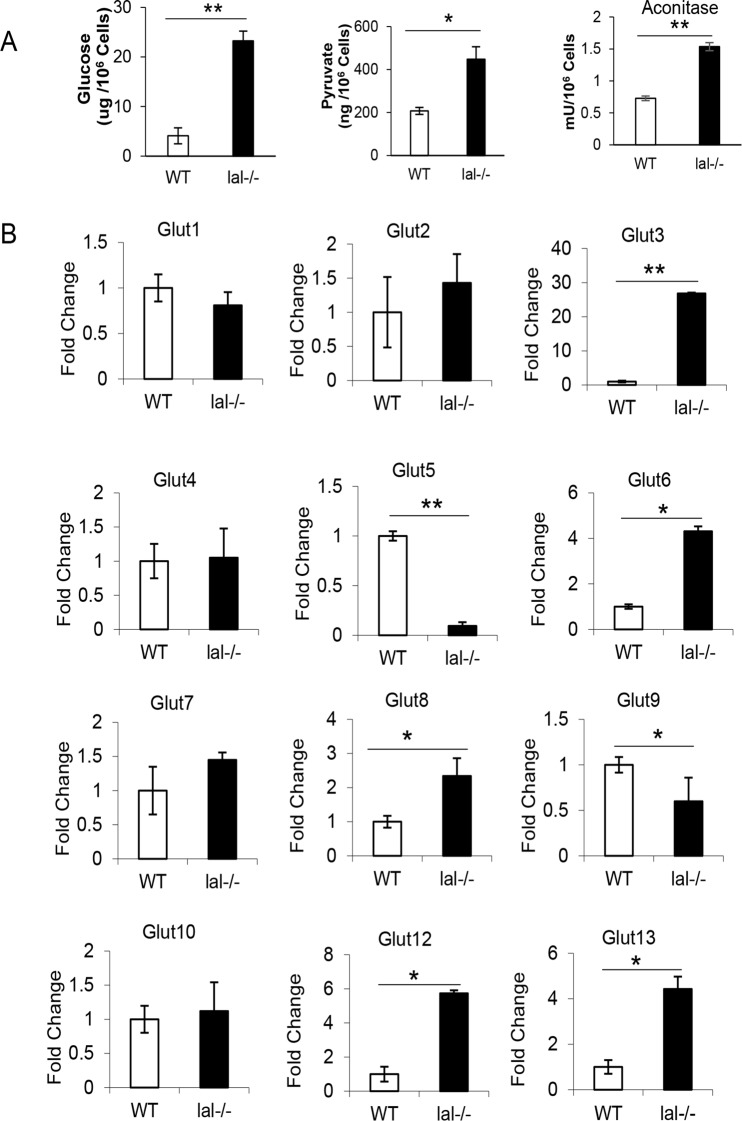
Glucose transportation, glycolysis and TCA in HD1A and HD1B cells. (**A**) The glucose and pyruvate concentrations, and aconitase activity in wild type (HD1A) and *lal*-/- (HD1B) cells. The results are means ± SD from three independent experiments (n = 3), *, p< 0.05, **, p< 0.001. (**B**) Expression of *Glut1 to Glut13* with the housekeeping gene β-*Actin* as internal control by Real-time PCR. The results are means ± SD from three independent experiments (n = 3), *, p< 0.05, **, p< 0.001.

Compared with HD1A cells, HD1B cells showed increased glucose concentration (Fig. 2A), suggesting the enhanced glycolysis metabolic pathway, in which glucose converts into pyruvate. Indeed, the pyruvate concentration was increased in HD1B cells compared with that in HD1A cells (Fig. 2A). Glycolysis occurs in the cytosol of the cell. Pyruvic acid supplies energy to living cells through the citric acid cycle (TCA) in the mitochondria, which generates NADH for the oxidative phosphorylation (OXPHOS, electron transport pathway) to produce ATP. Aconitase is the rate-limiting enzyme in the TCA cycle. Its activity was doubled in HD1B cells compared with HD1A cells (Fig. 2A). The high glycolysis metabolic rate and TCA turnover promoted us to investigate GLUT (SLC2) family members [22]. These are the major membrane transporters. Among them, GLUT 1–5 have been well characterized as glucose and/or fructose transporters in various tissues and cell types. Thirteen GLUT proteins have been reported to be expressed in mice (14 in humans). Using the Real-time PCR method, expression of all GLUT members was assessed in HD1A and HD1B cells, in which GLUT3, GLUT6, GLUT8, GLUT12, and CLUT13 were upregulated, while GLUT 5 and GLUT 9 were downregulated in HD1B cells (Fig. 2B). This supports a concept that the neutral lipid metabolic pathway controls the balance of glucose transportation to fuel the energy need in myeloid cells.

### Mitochondrial morphology change in HD1B cells

**Fig 3:**
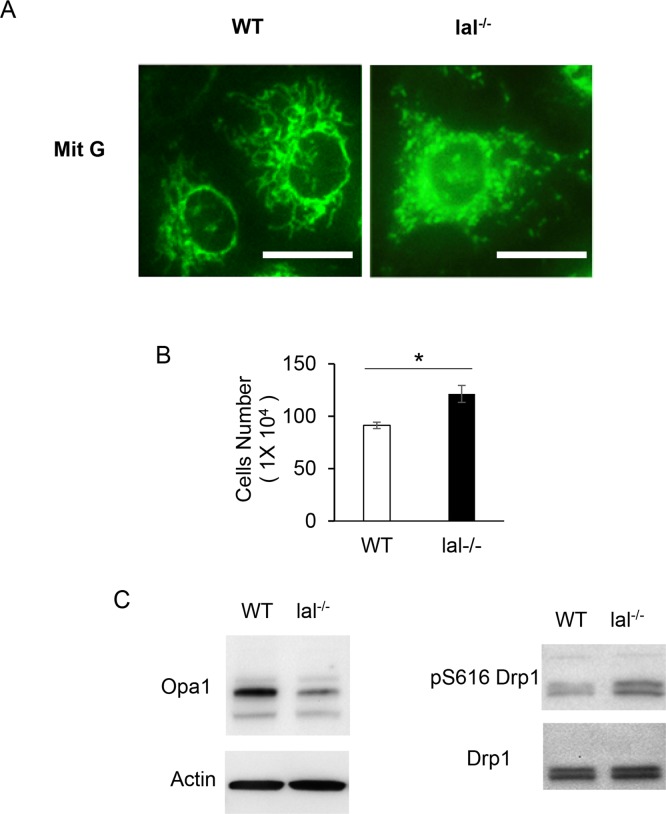
The morphological change of mitochondria in wild type (HD1A) and *lal*-/- (HD1B) cells. (A) MitoTracker Green FM staining of live wild type (HD1A) and *lal*-/- (HD1B) cells. Bar = 25 μm. (B) Cell proliferation of wild type (HD1A) and *lal*-/- (HD1B) cells *in vitro*; (C) Western blot analyses of protein expression of Opa1 and phosphorylation at Ser616 of DRP1 in wild type (HD1A) and *lal*-/- (HD1B) cells. (D) Concentrations of glucose, pyruvate and the aconitase activity in HD1A and HD1B cells were measured. Results are means ± SD from three independent experiments (n = 3), *, p< 0.05.

As demonstrated above, compared to HD1A cells the glycolytic pathway in HD1B cells are enhanced and utilized to fuel the energy need for this transition. Mitochondria are central organelles in carbohydrate, lipid and amino acid metabolisms in cells. Glycolytic metabolic influx into mitochondria and enter the TCA cycle to enhance mitochondrial respiration on the mitochondrial electron transport chain (ETC). Mitochondria are double-membrane—bound subcellular organelles of eukaryotic cells with various functions, including oxidative-phosphorylation, apoptosis, and ROS production. In order to fully understand pathogenic malformation and malfunction in HD1B cells, it is necessary to characterize and compare mitochondrial structures in HD1A and HD1B cells for comparison. It has been well documented that the mitochondrial fission and fusion processes play critical roles in governing these mitochondrial functions [19,23]. Using mitochondria-specific labeling dye Mit G, mitochondria in HD1B cells showed more fission shaped structure (dots) when compared with more fusion shaped structure in HD1A cells (Fig. 3A). The fission process of mitochondria is engaged in a more proliferative state [19,23]. Indeed, HD1B cells are more proliferative than HD1A cells (Fig. 3B). This observation is also in consistence with higher transportation and consumption of glucose as outlined in Fig. 1. Opa1 is a key mediator controlling mitochondrial fusion. Western blot analysis showed a decreased level of Opa1 expression in HD1B cells compared with that of HD1A cells (Fig. 3C, left panel). On the other hand, Drp1 is a key mediator controlling mitochondrial fission. Phosphorylation on Ser616 activates Drp1 to stimulate mitochondrial fission. In HD1B cells, phosphorylation on Ser616 of Drip1 was increased significantly (Fig. 3C, right panel). Therefore, LAL deficiency in HD1B cells leads to mitochondrial fusion to fission conversion, a more proliferative state.

### Mitochondrial dysfunction in HD1B cells

**Fig 4:**
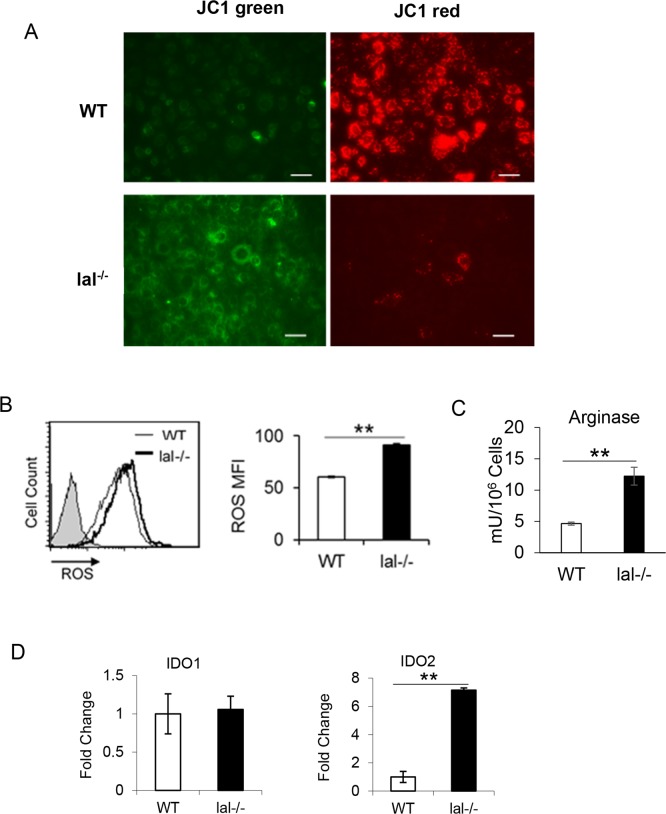
Mitochondrial membrane potential, ROS production, arginase, and IDOs in wild type (HD1A) and *lal*-/- (HD1B) cells. (A) Live wild type (HD1A) and *lal*-/- (HD1B) cells were stained with JC1 to measure the mitochondria membrane potential. JC1 fluorescent red staining represents a healthy membrane potential state, while JC1 fluorescent green staining represents a damaged membrane potential state. (B) The ROS levels in wild type (HD1A) and *lal*-/- (HD1B) cells by flow cytometry analysis. Results are mean ± SD from three independent experiments (n = 3), **, p< 0.001. (C) The arginase activity in HD1A and HD1B were measured. The result is mean ± SD from three independent experiments (n = 3), **, p< 0.001. (D) The IDO1 and IDO2 expression levels were measured by Real-Time. The results are means ± SD from three independent experiments (n = 3), **, p< 0.001.

To see if the structural change leads to functional changes, the mitochondrial membrane potential and ROS production, which are coupled with OXPHOS, were measured in HD1A and HD1B cells. The mitochondrial membrane potential was analyzed by JC1 staining. While most HD1A cells were stained with red fluorescence staining (representing healthy mitochondria), HD1B cells were stained with less red fluorescence and more green fluorescence (representing damaged mitochondria) (Fig. 4A). A damaged mitochondrial membrane potential restricts electron flow and increases the leakage of electrons to form ROS through the electron transport chain. Indeed, increased ROS production in HD1B cells was observed (Fig. 4B). Compared with that in HD1A cells, HD1B cells also increased the arginase activity, which is another important characteristic parameter of MDSCs (Fig. 4C). These phenotypes resemble to what were observed in *lal*-/- MDSCs [15]. Because tryptophan (Trp) metabolizing enzyme indoleamine 2,3-dioxygenase (IDO) plays a pivotal role in MDSCs via suppressing T cell function [24], expression of both IDO1 and IDO2 were measured in HD1A and HD1B cells. Interestingly, only IDO2 was highly overexpressed in HD1B cells (Fig. 4D). Therefore, there are profound differences between the mitochondrial structures and functions in HD1A and HD1B cells.

### mTOR inhibition or antioxidant treatment rescued abnormal HD1B phenotypes

**Fig 5:**
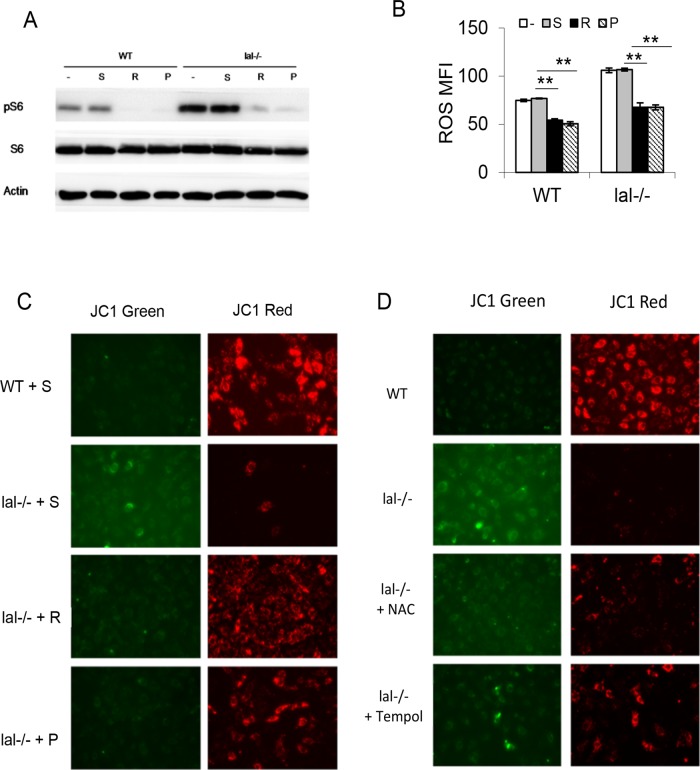
Over-activation of the mTOR signal pathway in wild type (HD1A) and *lal*-/- (HD1B) cells. (A) Western blot analyses of the phosphorylation level of mTOR downstream effector pS6 in wild type (HD1A) and *lal*-/- (HD1B) cells. Both cells were treated with solvent (S) or with mTOR inhibitor rapamycin (R) or PP242 (P). (B) Flow cytometry analyses of the ROS levels of rapamycin or PP242 treated or untreated wild type (HD1A) and *lal*-/- (HD1B) cells. Results are mean ± SD from three independent experiments (n = 3), p< 0.001. (C) Mitochondria membrane potential was analyzed in rapamycin or PP242 treated or untreated wild type (HD1A) and *lal*-/- (HD1B) cells by JC1 staining. Treatment of mTOR inhibitors restored the mitochondria membrane potential in *lal*-/- (HD1B) cells. (D) Antioxidant reagent NAC or Tempol treated or untreated wild type (HD1A) and *lal*-/- (HD1B) cells were stained with JC1 to measure the mitochondria membrane potential. Treatment of antioxidants restored the mitochondria membrane potential in *lal*-/- (HD1B) cells.

Previously, Affymetrix GeneChip microarray showed over-activation of the mTOR signaling pathway in association with mitochondrial membrane potential damage and ROS over-production in *lal*-/- MDSCs [15]. To see if mTOR is overly activated in HD1B cells, rapamycin or PP242 were used to suppress the mTOR signaling pathway. As demonstrated by the Western blot result, both inhibitors blocked mTOR downstream effector S6 phosphorylation in HD1A and HD1B cells (Fig. 5A). The treatment also decreased ROS production (Fig. 5B) and reversed the damaged mitochondrial membrane potential in HD1B cells (Fig. 5C). To confirm that ROS over-production is responsible for the damage of the mitochondrial membrane potential in HD1B cells, antioxidant NAC or TEMPOL was used to diminish ROS. This treatment partially improved the condition of the mitochondrial membrane potential as shown with more red JC-1 fluorescence staining in HD1B cells (Fig. 5D).

### Immunosuppressive function of HD1B cells

**Fig 6:**
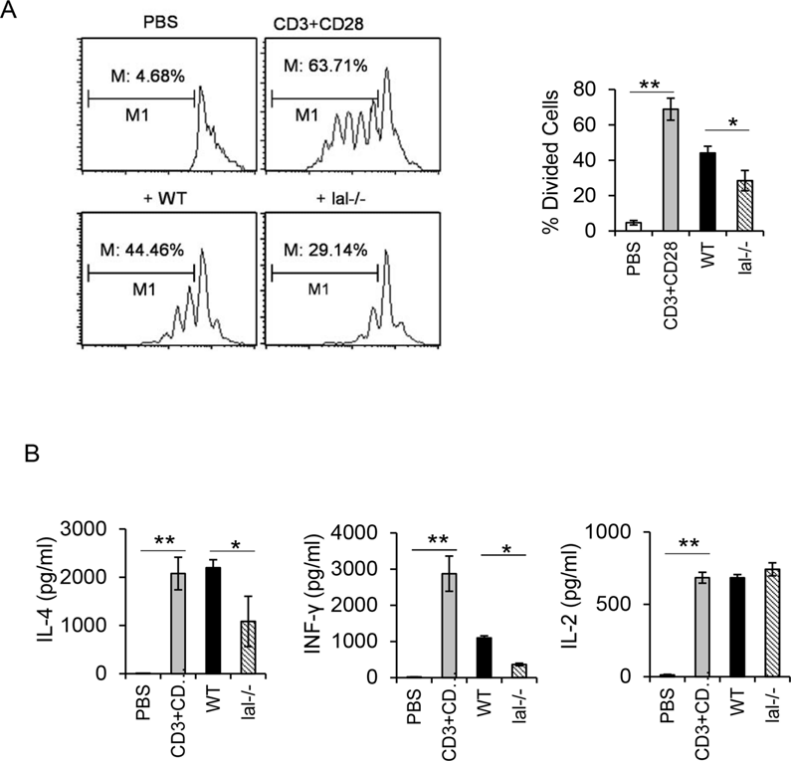
Immunosuppression on T cell proliferation and function by wild type (HD1A) and *lal*-/- (HD1B) cells. (A) CFSE labeled wild type CD4^+^ T cells were stimulated with anti-CD3 and anti-CD28 antibodies, and co-cultured with wild type (HD1A) or *lal*-/- (HD1B) cells (1:30). CD3 and CD28 antibody unstimulated CD4^+^ T cells served as a negative control. Results are mean ± SD from three independent experiments (n = 3), *, p< 0.05; **, p<0.01. (B) Secretion of T cell releasing IL-2, IL-4 and INF-γ in the above co-culture experiment was measured to assess the CD4^+^ T cell function. Results are mean ± SD from three independent experiments (n = 3), *, p< 0.05; **, p<0.01.

Immunosuppression is the hallmark of MDSCs. To see if HD1B cells possess immunosuppressive function, HD1B cells (or HD1A cells as control) were co-cultured with CFSE-labeled splenocyte CD4^+^ T cells in the anti-CD3 and anti-CD28 antibody coated plate. After 4 days, CD4^+^ T cell proliferation was analyzed by flow cytometry. Compared with HD1A cells, HD1B cells showed a stronger suppressive function on CD4^+^ T cell proliferation (Fig. 6A). Lymphokine release of INFγ (TH1) and IL-4 (TH2) by CD4^+^ T cells was also decreased when CD4^+^ T cells were co-cultured with HD1B cells (Fig. 6B). These results indicate that HD1B cells exhibit a similar immunosuppressive function as *lal*-/- MDSCs do.

### mTOR signal inhibition reversed HD1B cell immunosuppressive function

**Fig 7:**
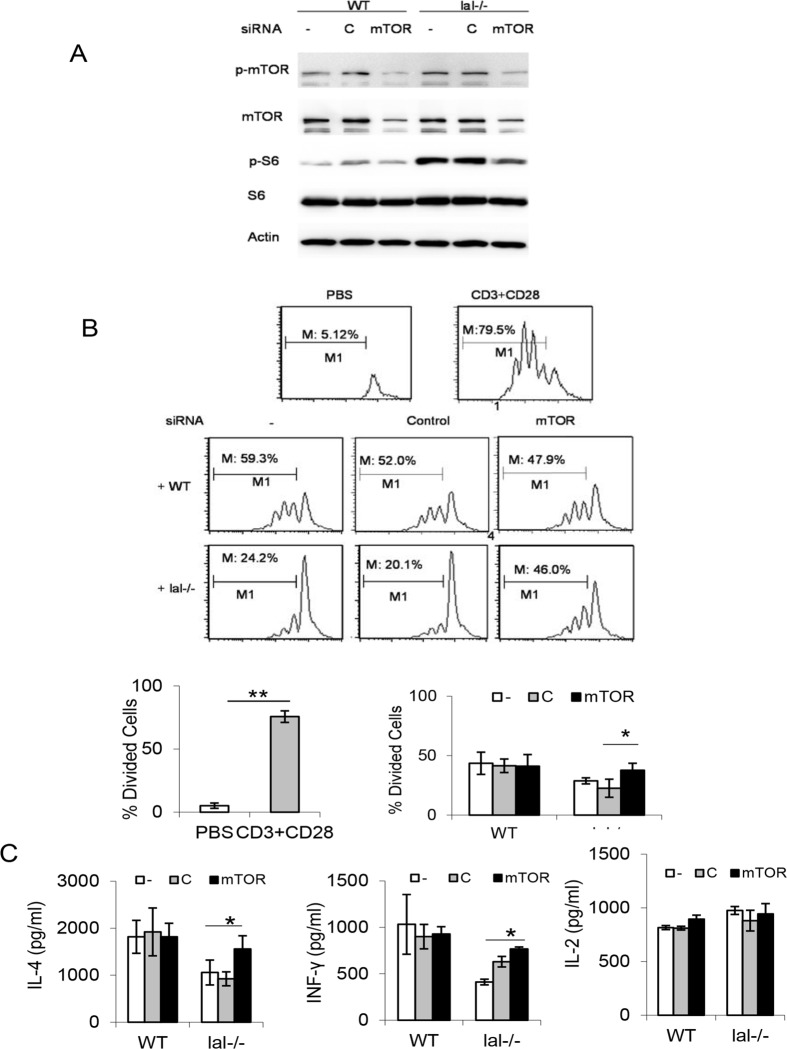
siRNA knockdown of mTOR inhibits immunosuppression on T cell proliferation and function of *lal*-/- (HD1B) cells. (A) Western blot analyses showed the mTOR protein expression level, phosphorylated mTOR, and phosphorylated S6 levels reduced in wild type (HD1A) and *lal*-/- (HD1B) cells by mTOR siRNA knocking down. (B) T cell suppressive activity of HD1B was reduced upon mTOR knocking down by siRNA transfection. Wild type (HD1A) or *lal*-/- (HD1B) cells were pretreated with control (C) siRNA or mTOR (mTOR) siRNA. Treated cells were incubated with CFSE labeled wild type CD4^+^ T cells and stimulated with anti-CD3 and anti-CD28 antibodies. Results are mean ± SD from three independent experiments (n = 3), p< 0.05; (C) Secretion of T cell releasing IL-4 in the above co-culture experiment was measured to assess the CD4^+^ T cell function. Results are mean ± SD from three independent experiments (n = 3), p< 0.05.

mTOR protein expression was knocked down by mTOR siRNA in HD1B cells as confirmed by Western blot analysis, which led to decreased mTOR and S6 phosphorylation (Fig. 7A). After transfection with mTOR siRNAs, HD1B cells showed reduced immunosuppression on splenocyte CD4^+^ T cell proliferation in the co-culture experiment (Fig. 7B), while control siRNAs showed no reduced effect. Furthermore, lymphokine IL-4 and INFγ secretion was also recovered when CD4^+^ T cells were co-cultured with HD1B cells that had been knocked down by mTOR siRNA compared with those knocked down by control siRNA (Fig. 7C). Therefore, mTOR over-activation is partially responsible for HD1B cell immunosuppressive function.

### HD1B cells stimulate tumor cell proliferation

**Fig 8:**
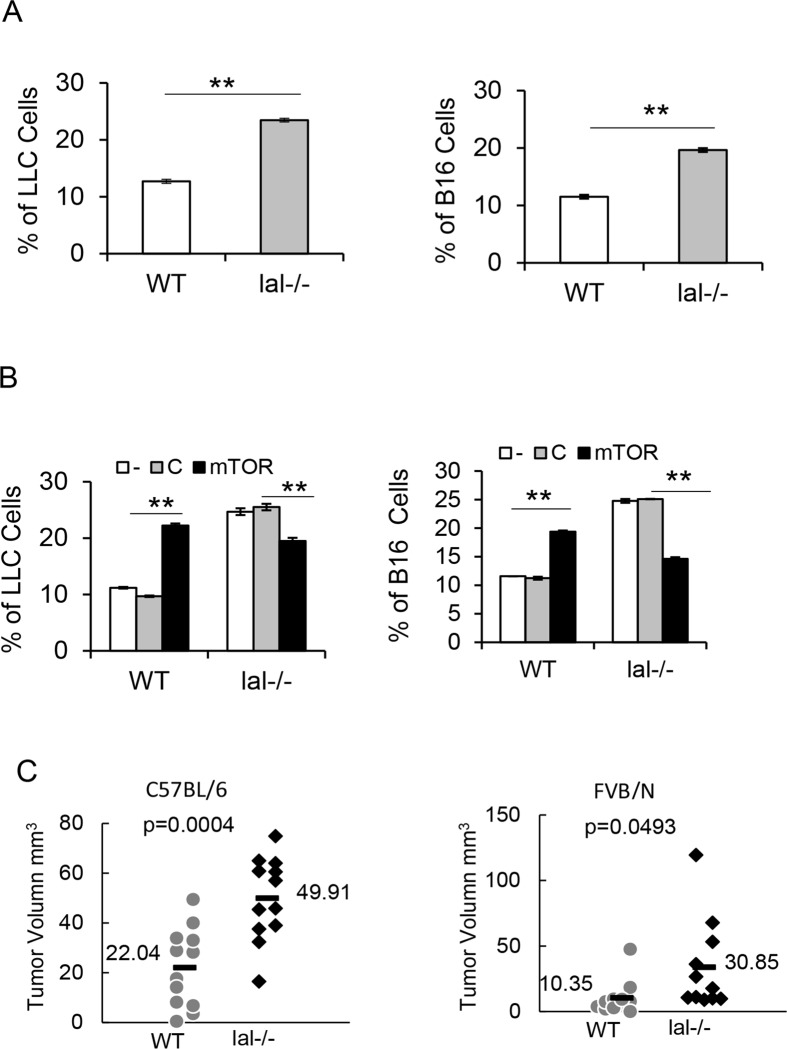
Lal-/- cells (HD1B) stimulated cancer cells growth. (A) Wild type (HD1A) and *lal*-/- (HD1B) cells were co-cultured with CFSE labeled LLC or B16 melanoma cells (5:1) for 3 days. The results are presented as percentage of CFSE positive cells increased from none co-culture base line. The results are mean ± SD from three independent experiments (n = 3), **, p< 0.01. (B) mTOR siRNA knockdown in *lal*-/- (HD1B) cells inhibited the stimulatory activity of cancer cell growth (LLC and B16 melanoma cells). The same cell ratio was used. The results are mean ± SD from three independent experiments (n = 3), **, p< 0.01; (C) B16 melanoma cells (2 X 10^5^) that were mixed with the same amount of wild type (HD1A) or *lal*-/- (HD1B) cells were left-side and right-side pair-injected subcutaneously into C57BL/6 or FVB/N mice. After 14 days, tumor volumes were measured using the formula: (length X width^2^) /2. The results are mean ± SD from 11–12 independent experiments (n = 11–12), p values are listed in comparison of tumor sizes between co-injection of wild type (HD1A) and *lal*-/- (HD1B) cells.

Recently, *lal*-/- MDSCs have been shown to directly stimulate cancer cell proliferation [11]. To compare the tumor stimulatory effects between HD1A and HD1B cells, both cells were co-cultured with CFSE-labeled B16 melanoma cells or LLC cells *in vitro*. After 3 days, more tumor cells were observed when co-cultured with HD1B cells compared with those co-cultured with HD1A cells in flow cytometry analysis (Fig. 8A), suggesting that HD1B cells possess a stronger stimulatory effect on both B16 melanoma cells and LLC cells. Knockdown of mTOR in HD1B cells showed a decreased stimulatory effect on cancer cells (Fig. 8B). Surprisingly, knockdown of mTOR in HD1A cells showed an increased stimulatory effect on cancer cells (Fig. 8B). Perhaps the mTOR signaling plays differential roles in HD1A cells and HD1B cells. In an in vivo model, when B16 melanoma cells were co-injected with HD1A or HD1B cells into syngeneic C57BL/6 or allogeneic FVB/N wild type recipient mice, the tumor size and volume were much larger in the group with HD1B cell co-injection than the group with HD1A cell co-injection in both syngeneic and allogeneic background (Fig. 8C).

## Discussion

MDSCs are heterogeneous immune cell populations involved in various pathogenic diseases. Although it is doable, isolation of MDSCs from mice and humans is time consuming and costly. Therefore, there is an urgency to establish an “MDSC-like” cell line for various biochemical, immunological, physiological, pathological and pharmacological studies. Establishment of such cell lines will greatly facilitate characterization of MDSCs biological functions. It will also greatly benefit screening and discovery of new pharmacological drugs, as well as identifying new immune therapeutic approaches to target MDSCs.

Lipids have long been recognized not only as the nutrients for cell growth and structural components, but also as the cell signaling molecules that have the capacity to trigger profound physiological responses. Previously, we have done extensive studies to show that LAL is a critical enzyme that controls inflammation, especially MDSCs development and homeostasis. Deletion of this neutral lipid metabolic controlling enzyme induces massive MDSCs expansion in *lal*-/- mice, which leads to immunosuppression and multiple pathogenic diseases including cancer formation and metastasis [5–7,9,11–15,25]. These studies have firmly established the functional roles of neutral lipid metabolism controlled by LAL in MDSCs development, homeostasis and function. Based on these solid characterizations, *lal*-/- mouse model is an ideal system to generate “MDSCs-like” cell lines. By crossbreeding wild type or *lal*-/- mice with Immortomouse expressing a temperature-sensitive version of simian virus 40 large T antigen [21], we have successfully established two myeloid-derived cell lines, HD1A (wild type myeloid cell line) and HD1B (*lal*-/- myeloid cell line).

In *lal*-/- HD1B cells, more intensified lysosomal subcellular structures were observed by both LysoTracker and LAMP1 staining studies compared with HD1A cells (Fig. 1B, C), implicating that LAL deficiency increases lysosome genesis. In addition, the lysosomal structures in HD1B cells were located around perinuclear areas, while lysosomal structures in HD1A were spread out in the cytoplasm. This localization change implicates functional changes of lysosomes in HD1B cells. The lysosome functions far beyond the traditional cell component to degrade and recycle cellular waste. It is also involved in much broader functions such as secretion, plasma membrane repair, cell signaling, autophagy and energy metabolism [26].

In addition to morphological changes, expression of several functional proteins critical for fatty acid transportation across the plasma membrane or mitochondrial membrane, and metabolic stress protein were investigated. CD36 is a scavenger receptor for modified LDL lipid particles and long-chain fatty acid uptake [27]. To compensate the inability of intracellular fatty acid generation due to LAL deficiency, HD1B cells upregulated CD36 expression perhaps trying to export more extracellular ones (Fig. 1D). On the other hand, due to the reduced intracellular generation of fatty acids in HD1B cells, there is no change in expression of carnitine *O*-palmitoyltransferase (CPT), the rate-limiting enzyme for long chain fatty acid entry into mitochondria and fatty acid oxidation (FAO) (Fig. 1D). However, under the metabolic stress, mTOR activated forkhead box protein O 3 (FOXO3) was upregulated in HD1B cells (Fig. 1D). When high levels of ROS are generated, FOXOs translocate into the nucleus to activate both lysosomal and proteasomal protein degradation. FOXO proteins activate the expression of genes that encode superoxide dismutase and catalase enzymes (required for the detoxification of ROS) [28,29]. It has also been shown that the transcription factor forkhead homeobox type protein O1 (FoxO1) is induced by nutrient restriction in adipocytes and exerts transcriptional control of lipid catabolism via the induction of LAL [30].

Co-regulators for histone acetyltransferases (HATs), including sirtuins (SIRTs) regulate the levels of FOXO acetylation during oxidative stress. NAD^+^-dependent SIRTs coordinate a switch from glucose to fatty acid oxidation during the acute inflammatory response [31]. At least SIRT1 expression remains unchanged, suggesting that the switch from glucose to fatty acid oxidation may not be needed in HD1B cells.

In the mitochondria, oxidative phosphorylation (OXPHOS) is the metabolic pathway to reform ATP by the oxidation of nutrients. There are several catabolic biochemical processes produce energy (in form of ATP), including glycolysis, the citric acid cycle, and β-oxidation of free fatty acids. In fat catabolism, triglycerides are hydrolyzed to break into fatty acids and glycerol by LAL. Fatty acids are further broken down through a process known as β-oxidation and results in acetyl-CoA, which can be used in the citric acid cycle in mitochondria. β-oxidation of fatty acids with an odd number of methylene bridges produces propionyl CoA, which is converted into succinyl-CoA and fed into the citric acid cycle. In the absence of the regular supply of fatty acids during LAL deficiency, it seems that the energy consumption switches more to the metabolic pathway on extracellular glucose consumption to fuel OXPHOS in HD1B cells. This was first observed by gene microarray analysis, in which the glycolytic metabolic gene profile of bone marrow *lal*-/- MDSCs is increased [15]. Glycolysis breaks glucose (a six-carbon-molecule) down into pyruvate (a three-carbon molecule). Pyruvate moves into the mitochondria to be converted into acetyl-CoA by decarboxylation and enters the citric acid cycle. As demonstrated in Fig. 2, both glycolysis (measured by the pyruvate concentration) and TCA in mitochondria (measured by the aconitase activity) were significantly elevated in HD1B cells. This observation was further supported by increased expression of glucose transporters (GLUT3, GLUT6, GLUT8, GLUT12, and CLUT13) in HD1B cells (Fig. 2). On the other hand, there was no difference between expression levels of CPT1a, CPT1b and CPT1c that are required for transporting long-chain FA into the mitochondria in HD1A cells and HD1B cells (Fig. 1D). There is a report showing that in IL-15 memory T cells, glucose is used to produce FA for OXPHOS, which is dependent on LAL to funnel fatty acids into mitochondria for oxidative phosphorylation [32]. This is unlikely in HD1B cells.

We previously reported that Affymetrix GeneChip microarray of *lal*-/- bone marrow Ly6G^+^ cells (almost all bone marrow Ly6G^+^ cells are CD11b^+^ in *lal*-/- mice) revealed over-activation of the mTOR signaling pathway [15]. Pharmacological inhibition of mTOR blocked *lal*-/- CD11b^+^Ly6G^+^ cell development and expansion [18]. It has been well documented that the mTOR signaling controls mitochondrial functions, including maintaining proper membrane potential and ROS production. ROS are generated as by-products of aerobic respiration and various other catabolic and anabolic processes [33]. Mitochondria are the major producer of ROS in cells at the electron transport chain. Electrons leak from the electron transport chain directly to oxygen, producing short-lived free radicals. A decline in mitochondrial function such as damaged membrane potential leads to enhanced ROS production. Indeed, *lal*-/- Ly6G^+^ cells showed damaged mitochondrial membrane potential and increased ROS production [15]. These abnormal mitochondrial functions can be reversed by mTOR pharmacological inhibitors [18]. Similarly, the mTOR downstream gene S6 was hyper-phosphorylated in HD1B cells compared with that in HD1A cells (Fig. 5A), indicating over-activation of mTOR signaling in HD1B cells. Damaged mitochondrial membrane potential and increased ROS production were observed as well in HD1B cells (Fig. 4A, B). Similar to those observed in isolated *lal*-/- MDSCs, these abnormal mitochondrial activities can be blocked by treatment with mTOR pharmacological inhibitor rapamycin and PP242 (Fig. 5B and 5C), as well as by anti-ROS chemicals (Fig. 5D). Interestingly, the mitochondrial structure in HD1B cells showed a more fission pattern (dots), whereas HD1A showed a more fusion pattern (linear lines) morphologically by Mit G staining (Fig. 3A). Importantly, pro-fusion protein Opa1 was down-regulated, while phosphorylation of pro-fission protein Drp1 was increased in HD1B cells compared with HD1A cells (Fig. 3C). Mitochondria are double-membrane—bound subcellular dynamic organelles that constantly fuse and divide. Mitochondrial fission and fusion processes are essential for mitochondrial functions to meet the cellular activity of proliferation. Since the mitochondrial fission state indicates more cell proliferation, this is in agreement with a higher proliferative rate of isolated *lal*-/- MDSCs [15] and HD1B cells (Fig. 3B). Compared with that in HD1A cells, HD1B cells also increased the arginase activity, which is another important characteristic parameter for MDSCs [34], (Fig. 4C). Interestingly, IDO2 was highly overexpressed in HD1B cells, whereas IDO1 did not (Fig. 4D). It has been reported that high-fat feeding induces IDO2 in the liver [35].

Functionally, the hallmark feature of MDSCs is to suppress T cell proliferation and function. We have previously shown that *lal*-/- CD11b^+^Ly6G^+^ MDSCs exhibit strong immunosuppressive function on T cell proliferation and lymphokine secretion. This is partially responsible for the decreased T cell populations in *lal*-/- mice [12,14]. Therefore, it is essential to demonstrate that HD1B cells possess immunosuppressive function before claiming it as an “MDSC-like” cell line. When co-cultured with wild type CD4^+^ T cells in vitro and stimulated with anti-CD3 and anti-CD28 antibodies, HD1B cells showed a strong suppressive activity on CD4^+^ T cell proliferation (Fig. 6A), and TH1 INFγ, TH2 IL-4 secretion (Fig. 6B). After transfection with mTOR siRNAs, HD1B cells showed a decreased immunosuppressive activity on CD4^+^ T cell proliferation (Fig. 7B) and lymphokine secretion (Fig. 7C). This is also similar to what has been observed in *lal*-/- CD11b^+^Ly6G^+^ MDSCs [18]. The second hallmark for *lal*-/- MDSCs is their ability to directly stimulate cancer cell proliferation *in vitro* [11]. Interestingly and importantly, HD1B cells also demonstrated stimulatory activity on cancer cells both in *in vitro* co-culture experiment and in *in vivo* co-injection experiment, including LLC and B16 melanoma cancer cell models (Fig. 8).

In summary, several parallel studies showed resemblances between *lal*-/- MDSCs and the newly established MDSC-like HD1B cell line, including but not limited to: 1) Both *lal*-/- MDSCs and HD1B cells showed increased glycolytic metabolic activity; 2) Both *lal*-/- MDSCs and HD1B cells showed over-activation of mTOR signaling; 3) Both *lal*-/- MDSCs and HD1B cells showed the increased mitochondrial membrane potential damage and altered expression of metabolic molecules involved in mitochondrial functions; 3) Both *lal*-/- MDSCs and HD1B cells showed increased ROS production; 4) Both *lal*-/- MDSCs and HD1B cells showed immunosuppressive function on T cell proliferation and lymphokine secretion; 5) All above pathogenic cellular activities were corrected by either mTOR pharmacological inhibitors, or by siRNA knockdown in *lal*-/- MDSCs and HD1B cells; 6) Both *lal*-/- MDSCs and HD1B cells showed stimulation on cancer cell proliferation and growth. Taken all together, this newly established HD1B cell line shows similar characteristics of MDSCs from *lal*-/- mice, and support a concept that LAL supports FAO in myeloid cells and that lysosomal lipolysis contributes to normal function of myeloid cells.

In our characterization, one discrepancy between *lal*-/- MDSCs and HD1B cells is that HD1B cells are negative for CD11b and Ly6G, two hallmarks for MDSCs. Expression of cell surface markers (e.g. CD11b and Ly6G) depends on extracellular stimuli (e.g. cytokines, chemokines) in the surround environment in vivo. Since HD1A and HD1B are cell lines cultured in vitro, which are separated from the in vivo environment, CD11b and Ly6G negative expression may result from absence of stimulatory molecules for these two markers in the culture medium. This interesting observation indicates that CD11b and Ly6G may not be responsible for MDSCs malfunction. Therefore, to define an MDSC cell line, one has to rely on its functions, not a few cell surface markers. Nevertheless, both HD1A and HD1B cell lines will be useful in the future to explore the LAL functions in numerous cellular processes and functions of myeloid cells, especially MDSCs. The myeloid cell lines will also be useful for pharmacological identification of drugs to suppress MDSCs expansion and function in multiple inflammation-induced diseases which involve MDSCs.
